# Understanding seasonal weight loss tolerance in dairy goats: a transcriptomics approach

**DOI:** 10.1186/s12864-020-06968-2

**Published:** 2020-09-14

**Authors:** José Ricardo Parreira, Lorenzo Enrique Hernández-Castellano, Anastasio Argüello, Juan Capote, Noemí Castro, Susana de Sousa Araújo, André Martinho de Almeida

**Affiliations:** 1 IBET – Instituto de Biologia Experimental e Tecnológica, Av. da República, 2780-157 Oeiras, Portugal; 2 ITQB NOVA – Instituto de Tecnologia Química e Biológica António Xavier, Universidade Nova de Lisboa, Av. da República, 2780-157 Oeiras, Portugal; 3 Department of Animal Science, AU-Foulum, Aarhus University, 8830 Tjele, Denmark; 4 Animal Production and Biotechnology group, Institute of Animal Health and Food Safety, Universidad de Las Palmas de Gran Canaria, 35413 Arucas, Spain; 5 Unit of Animal Production, Pasture, and Forage in Arid and Subtropical Areas, Canary Islands Institute for Agricultural Research, 38270 La Laguna, Spain; 6 LEAF - Linking Landscape, Environment, Agriculture And Food, Instituto Superior de Agronomia, Universidade de Lisboa, Tapada da Ajuda, 13409-017 Lisbon, Portugal

**Keywords:** Goat, Mammary gland, Transcriptomics, RNA-sequencing, Seasonal weight loss

## Abstract

**Background:**

Seasonal weight loss (SWL) is a very important limitation to the production of ruminants in the Mediterranean and Tropical regions. In these areas, long dry seasons lead to poor pastures with low nutritional value. During the dry season, ruminants, particularly those raised in extensive production systems, lose around 30% of their body weight. Seasonal weight loss has important consequences on animal productive performance and health. In this study, RNA sequencing was used to characterize feed restriction effects in dairy goat of 2 breeds with different SWL tolerance: *Majorera* (tolerant) and *Palmera* (susceptible). Nine *Majorera* and ten *Palmera* goats were randomly distributed in a control and a restricted group: *Majorera* Control (adequately fed; MC; *n* = 4), *Palmera* Control (adequately fed; PC; *n* = 6), *Majorera* Restricted (feed restricted; ME; *n* = 5) and *Palmera* Restricted (feed restricted; PE; n = 4). On day 22 of the trial, mammary gland biopsies were collected for transcriptomics analysis.

**Results:**

From these samples, 24,260 unique transcripts were identified. From those, 82 transcripts were differentially expressed between MC and ME, 99 between PC and PE, twelve between both control groups and twenty-nine between both restricted groups.

**Conclusions:**

Feed restriction affected several biochemical pathways in both breeds such as: carbohydrate and lipid transport; intracellular trafficking, RNA processing and signal transduction*.* This research also highlights the importance or involvement of the genes in tolerance (*ENPP1*, *S-LZ*, *MT2A* and *GPNB*) and susceptibility (*GPD1*, *CTPS1*, *ELOVL6* and *NR4A1*) to SWL with respectively higher expression in the *Majorera* restriced group and the *Palmera* restricted group in comparison to the control groups. In addition, results from the study may be extrapolated to other dairy ruminant species.

## Background

Ruminant production plays an important role in the economy and livelihood of countries all across the world. Cattle and small ruminant production are particularly important in developing countries. Small ruminants, mainly sheep (*Ovis aries*) and goats (*Capra hircus*), are adapted to extensive production systems, particularly in drought-prone areas of the world. Such regions are characterized by the existence of dry seasons that affect growth, quality and availability of pastures, which directly impacts animal performance. Consequently, animals raised under extensive conditions are frequently subjected to feed restriction, a problem known as Seasonal Weight Loss or SWL [1].

Seasonal Weight Loss is considered one of the most significant problems for animal production worldwide. In fact, in regions such as the tropics or the Mediterranean basin, animals may lose up to 30% of their body weight (BW). Seasonal Weight Loss has been described in various regions of the globe such as West Africa [2, 3], South Africa [4, 5], Western Australia [6, 7] and the Canary Islands [8, 9]. The use of supplementation (either fodder, grain or concentrate feeds) is a possible solution to overcome SWL. Nevertheless, it is very costly and challenging, particularly in extensive production systems. Alternatively, the use of local breeds, known to be tolerant to SWL, is a very interesting and sustainable possibility for those regions as it is much less expensive and easy to implement in comparison to supplementation [1].

The Canary Islands (Spain) is a subtropical archipelago, with different climates across the islands. The western islands, namely *La Palma*, have a humid temperate climate. On the contrary, the easternmost islands, particularly *Fuerteventura* and *Lanzarote* have a dry climate [10]. These two different parts of the archipelago have in turn different rainfall that lead to differences in pasture availability and therefore in animal production systems. As a result of specific animal adaptations to the divergent climates of the islands, three local goat breeds are found. Goats from the Canary Islands descend from ancestors brought in from North Africa and Spain in the fifteenth and sisteenth centuries [11]. These breeds are the *Majorera* (from Gran Canaria and Fuerteventura), the *Palmera* from the island of La Palma and finally the *Tinerfeña* (from Tenerife island) [11]. *Majorera* goats have considerably high milk yields (500–550 kg of milk per lactation), being well adapted to dry regions with limited water and pasture availability and are therefore tolerant to SWL [8, 9]. On the contrary, *Palmera* goats are adapted to regions with higher rainfall, with better pastures, being susceptible to SWL [12]. We have demonstrated such different adaptation levels in previous work on productive and clinical biochemistry physiological parameters [8, 9].

The advent of Next Generation Sequencing (NGS) led to important developments in the field of animal science through the application of these techniques with different examples in reproduction and physiology found in the literature [13, 14]. In fact, and similarly to proteomics [15], numerous applications may be found in the literature, for mammary gland physiology, growth and involution, productive function, etc. [16].

Previous studies from our group have studied growth and productive traits [8] as well as different blood metabolites and hormone profiles [9] in *Majorera* (SWL tolerant) and *Palmera* (SWL susceptible) goat breeds subjected to feed restriction in order to simulate SWL. In such studies, significant differences between breeds were observed for blood metabolites (creatinine, urea, non-esterified fatty acids, cholesterol, IGF-1 and T3) as a consequence of underfeeding, highlighting major differences between breeds. We have recently extended that study to the effects of feed restriction on the mammary gland secretory tissue proteome [17, 18], metabolome [19] and fatty acid [20] profiling. We highlighted that the *Majorera* breed had a persistent accretion of proteins related to the immune system by comparison to *Palmera* goats [17]. Additionally, weight loss led in the *Palmera* breed to an accumulation of proteins related to apoptosis, overall confirming that the two goat breeds have different physiological reactions to SWL [17]. In this research, we hypothesize that differences observed between breeds and their response to feed restriction are reflected at the transcriptomic level. We therefore characterized the effects of feed restriction on the mammary gland secretory tissue transcriptome using an RNA sequencing (RNA-Seq) approach. The results highlighted major genes and biochemical pathways affected by weight loss in the two breeds.

## Results

### Goat body weights and productive performance

Fluctuations in body weight (BW) and milk yield in both breeds (*Majorera* and *Palmera*) under both conditions (feed restriction and control) were previously described and discussed [8]. Briefly, both control groups showed similar milk yields and slight weight fluctuations throughout the trial. Animals from the feed restricted groups showed decreased milk yields (86.9 and 87.2% reduction in the *Majorera* and *Palmera* breeds, respectively) and decreased 13% of their initial body weight at the end of the trial.

### Global overview of gene expression

Twelve RNA-seq libraries representing biological triplicates of studied experimental conditions were constructed and sequenced to capture transcriptome changes associated with the different breeds and feed regimes.

The sequencing of the 12 cDNA libraries resulted in a total of 700,489,334 raw reads, with an average of 58,374,111.17 raw reads/sample. Information about the library characterization for all samples can be found in Table 1.

**Table 1:** Individual characterization of the constructed RNA-seq libraries

Sample	Number of total bases	Number of raw reads	GC (%)	Q20 (%)	Q30 (%)	Number of mapped reads	Mapped reads (%)
MC1	5,513,718,472	54,591,272	48.78	95.44	91.40	38,107,784	70
MC2	6,080,971,034	60,207,634	50.15	93.42	88.97	37,251,096	62
MC3	5,746,342,278	56,894,478	49.59	94.41	90.06	36,979,098	65
PC1	5,016,985,322	49,673,122	50.11	93.90	89.47	29,401,920	59
PC2	5,328,847,668	52,760,868	49.3	94.97	90.84	35,422,448	67
PC3	5,462,951,832	54,088,632	49.72	94.48	90.17	30,622,836	57
ME1	5,440,414,692	53,865,492	48,46	94.74	90.68	36,580,712	68
ME2	6,052,955,250	59,930,250	47.71	94.75	90.84	41,953,734	70
ME3	5,943,007,458	58,841,658	48.44	95.30	91.30	40,345,942	69
PE1	7,931,399,306	78,528,706	49.27	95.06	91.03	54,606,756	70
PE2	6,455,995,144	63,920,744	48.33	94.62	90.57	45,854,954	72
PE3	5,775,834,278	57,186,478	48.66	95.13	91.06	38,565,456	67

MC *Majorera* Control, PC *Palmera* Control, ME *Majorera* Restricted, PE *Palmera* Restricted. Total bases, raw and mapped reads, GC content (%), Q20 (%) and Q30 (%) of each sequenced sample on the Illumina Hiseq 2500 (Illumina, San Diego, CA, USA) are described. Percentage of mapped reads was calculated using the number of mapped reads/raw reads

**Fig. 1:**
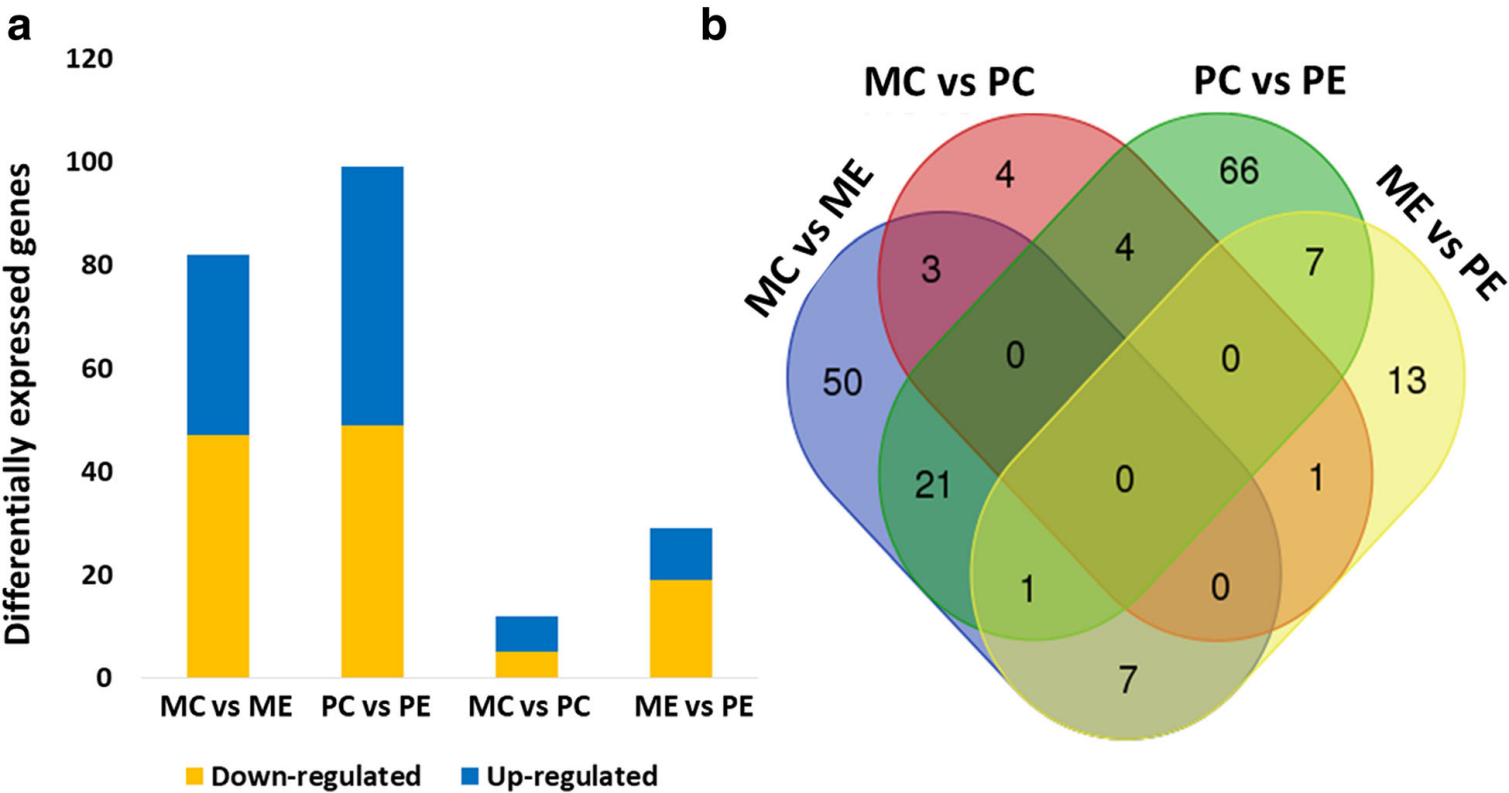
Differential gene expression results. **a** - The number of differentially (up and down-regulated) expressed genes (DEGs) in each comparison established for the study. **b** - Venn diagram analysis of DEGs in each comparison established for the study. The overlapping regions show common genes among the main comparisons established. MC – *Majorera* Control ME - *Majorera* Restricted; PC - *Palmera* Control and PE - *Palmera* Restricted. Parameters used: *P*-value and FDR < 0.05, Log2FC > 1.5 or < − 1.5, and raw number counts> 100 in at least one sample

Of the 24,260 genes found as output of the RSEM analysis, 9731 genes were found expressed amongst all samples (raw number counts > 100 in at least one sample) (Supplementary Material 1). Casein beta (*CSN2*), casein kappa (*CSN3*), progestagen-associated endometrial protein (*PAEP*) and α2-casein (*CSN1S2*) were among the identified sequences with highest total raw read counts, suggesting no ribosomal RNA (rRNA) contamination during library preparation. In addition, 82 transcripts showed significant changes in gene expression abundance (Log_2_FC <  -1.5 or Log_2_FC  >  1.5 −, *P* < 0.05 and FDR < 0.05) between MC (*Majorera* control) and ME (*Majorera* restricted), 99 between PC (*Palmera* control) and PE (*Palmera* restricted), 12 between both control groups and 29 between both restricted groups (Fig. 1). Some of the identified genes were differentially expressed across the different comparisons. These results are detailed in Supplementary materials 2, 3, 4, and 5.

**Fig. 2:**
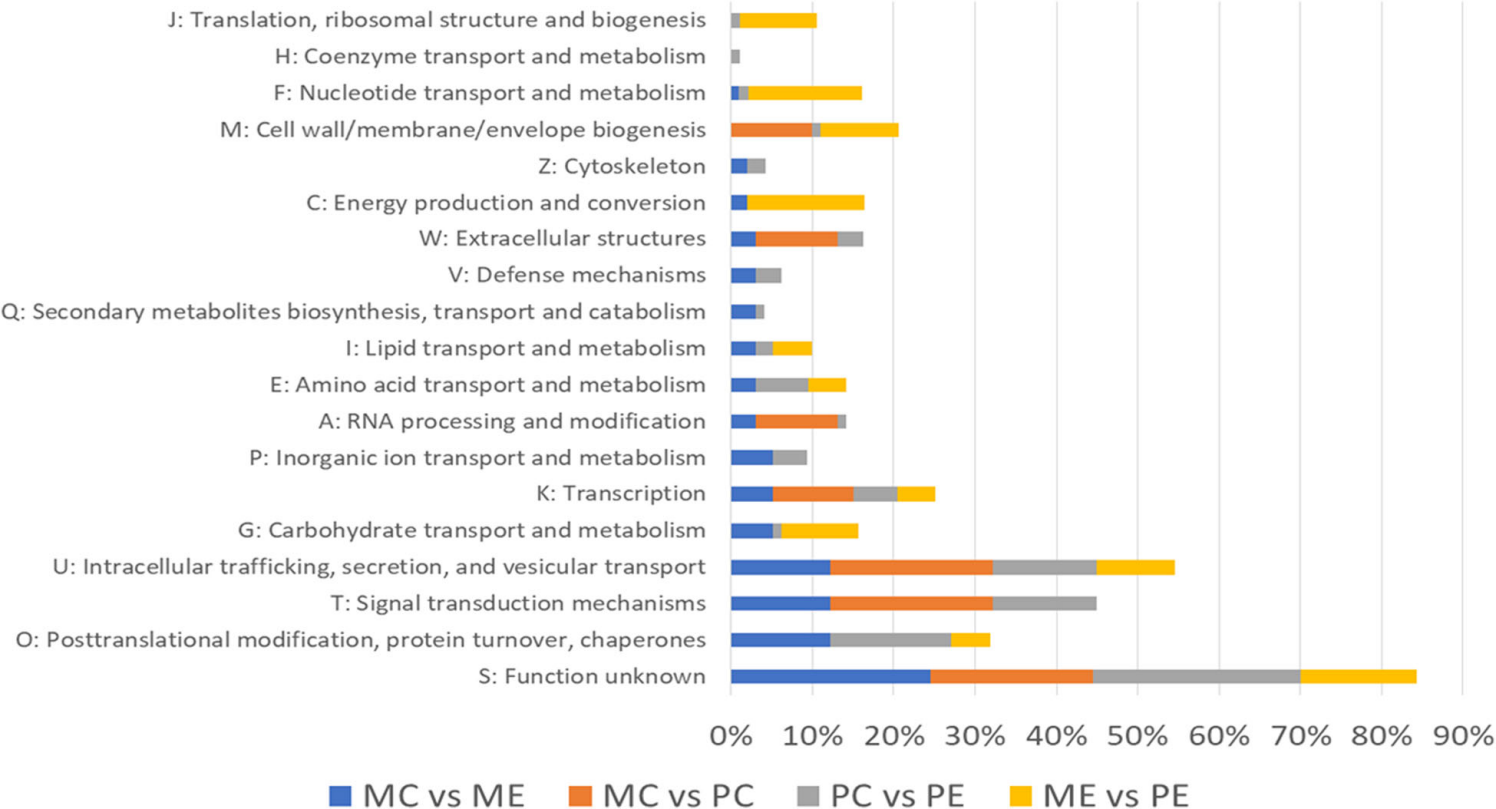
Graphic representation of the percentage of genes within each orthologous group obtained via Blast2GO, Cluster of orthologs e-Value 1e-3 for the four studied comparisons. MC – *Majorera* Control, PC – *Palmera* Control; ME – *Majorera* Restricted and PE – *Palmera* Restricted. For all comparisons: *p*-value and FDR < 0.05, log2FC > 1.5 or < − 1.5, and raw number counts> 100 in at least one sample

Cluster of Gene Ontology (COG)-based categorization describes the major functional classification regarding genes whose expression was trigggered in response to feed restriction in both breeds. An analysis of the differentially expressed genes (DEGs) in the four experimental groups (MC, PC, ME and PE) using the COG database is shown in Fig. 2. More details are available in Supplementary Material 7. For the two *Majorera* groups, sixteen COG terms were retrieved. These included for instance transcription, intracellular trafficking, carbohydrate transport and metabolism or signal transduction among other functions. For the *Palmera* groups comparison, eighteen COG terms were found including posttranslational modification and protein turnover, aminoacid transport and metabolism or defense mechanism functions. The comparison of the two control groups involved seven COG terms (e.g. RNA processing and modification or signal transduction mechanisms functions). Finally, the comparison of the two restricted groups led to eleven COG terms that included for instance nucleotide transport and metabolism, translation or energy production and conversion functions.

**Fig. 3:**
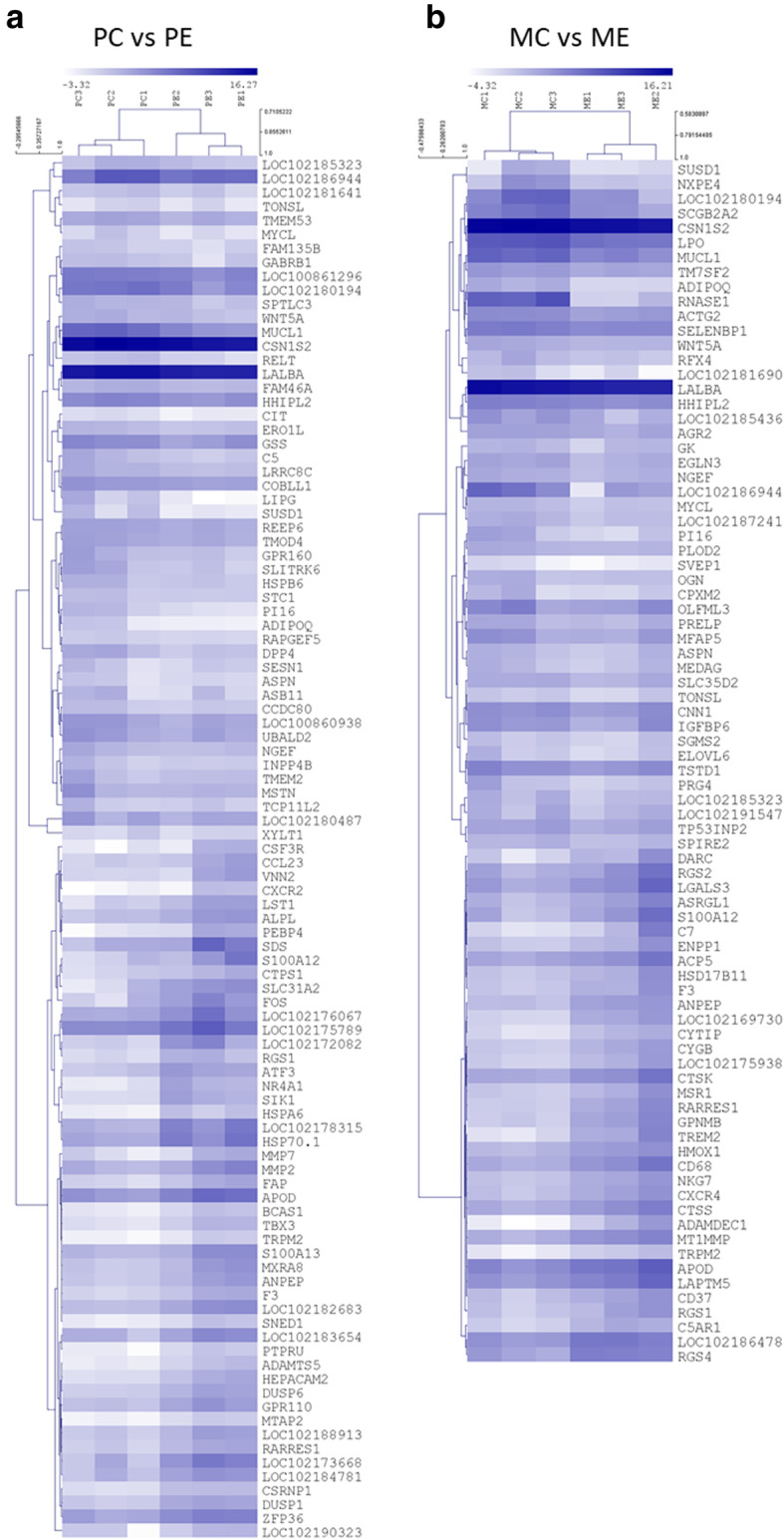
Heatmaps and hierarchical clustering of the differentially expressed genes in the *Palmera* (**a** panel) and the *Majorera* (**b** panel) animals. MC – *Majorera* Control; PC – *Palmera* Control; ME – *Majorera* Restricted and PE – *Palmera* Restricted. Gene symbols are displayed in rows. Dark blue indicates high expression whereas light blue indicates low expression

**Fig. 4:**
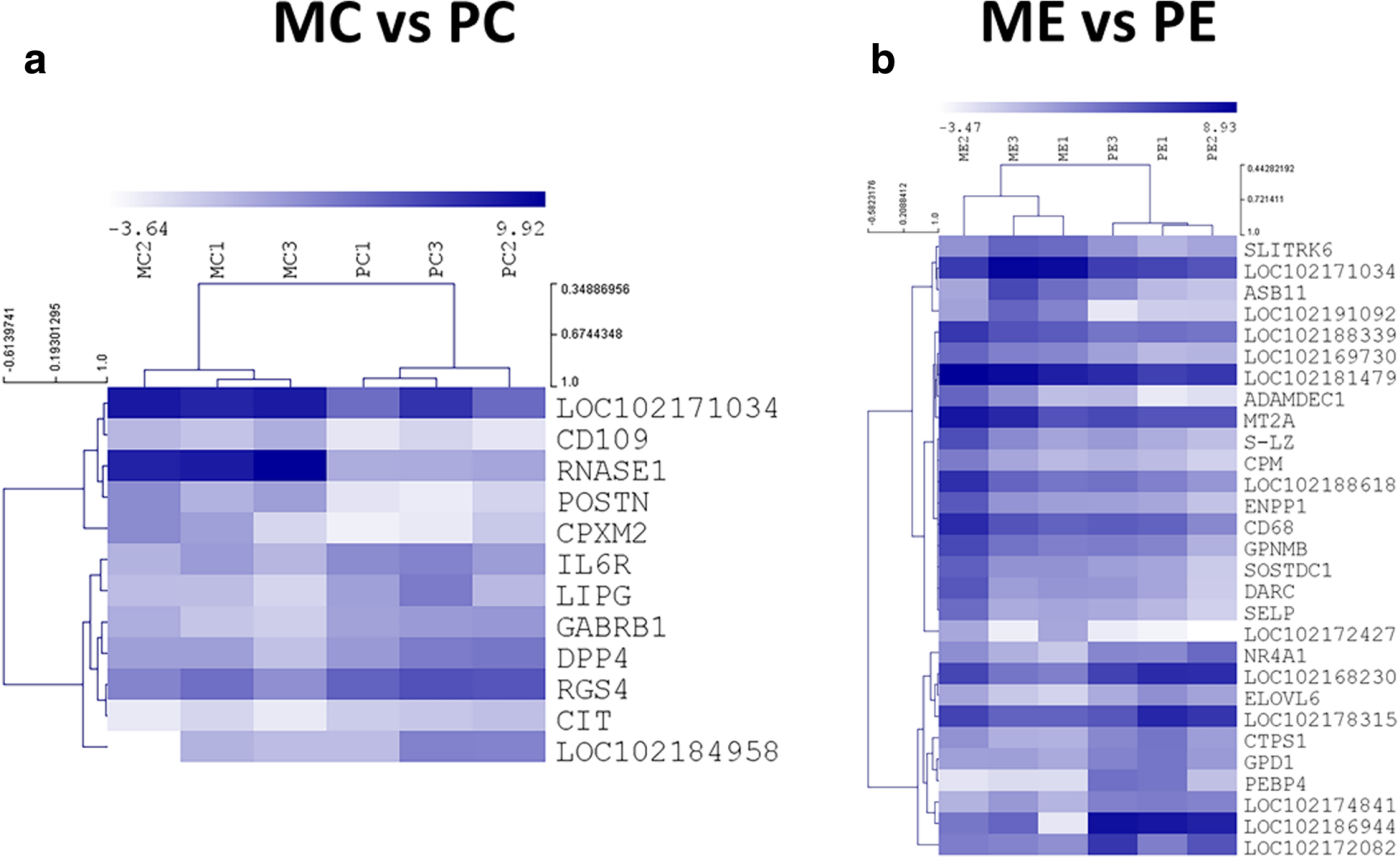
Heatmaps and hierarchical clustering of differential abundance transcripts in Control (**a** panel) and Restricted (**b** panel) animals. MC – *Majorera* Control; PC – *Palmera* Control; ME – *Majorera* Restricted and PE – *Palmera* Restricted. Gene symbols are displayed in rows. Dark blue indicates high expression whereas light blue indicates low expression

**Fig. 5:**
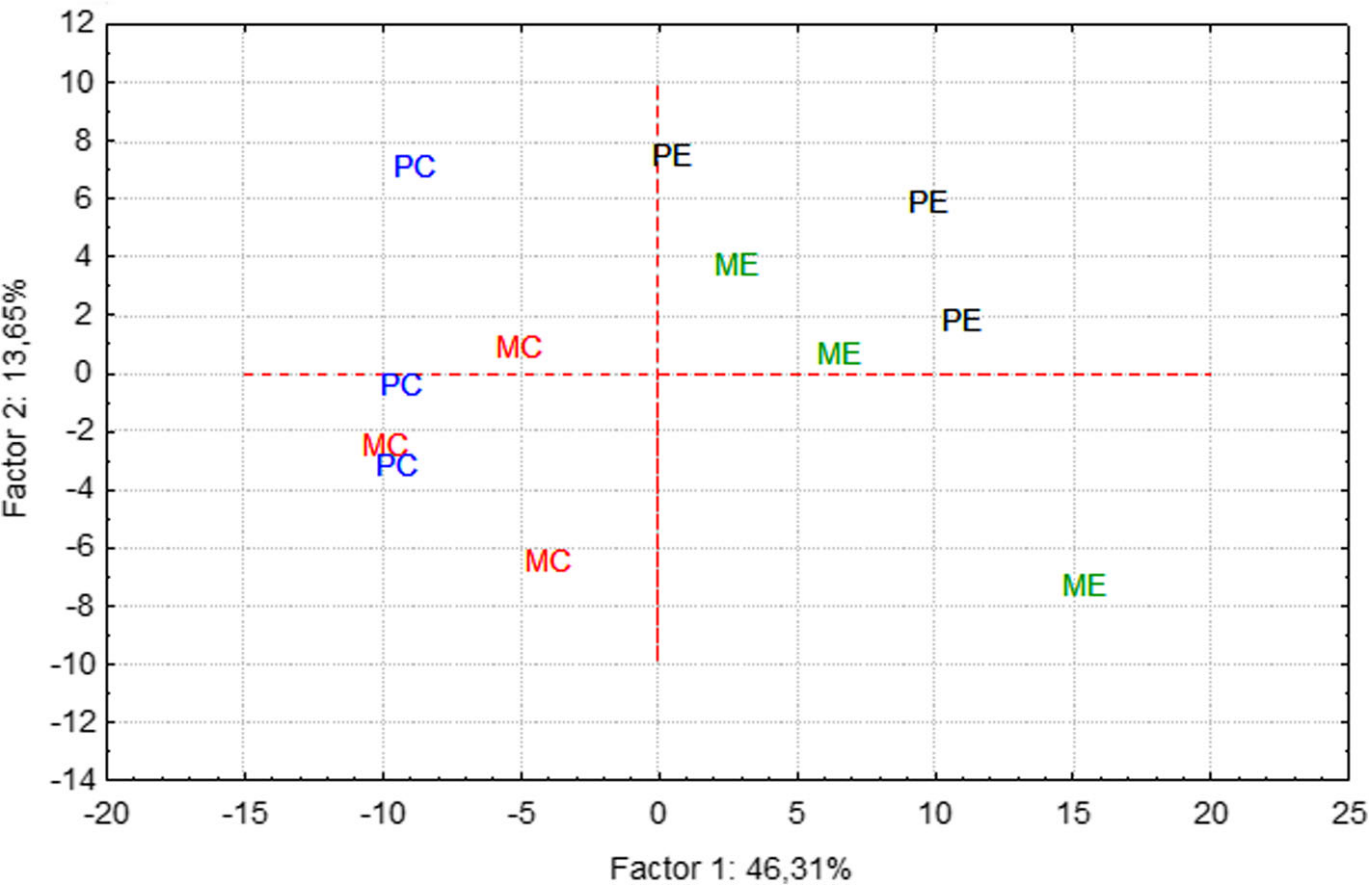
Principal Component Analysis (PCA) scatterplot of the four experimental groups using all  differentially expressed genes. Values of FPKM were Log_2_ transformed before computation. MC – *Majorera* Control; PC – *Palmera* Control; ME – *Majorera* Restricted and PE – *Palmera* Restricted

Hierarchical clustering made using the Log_2_ FPKM (fragments per kilo base per million mapped reads) values illustrate that all animals belonging to the same experimental condition are clustered together (Figs. 3 and 4). These show groups of genes that have differential expression in one of the pairwise comparisons. Examples include for instance, genes *POSTN* and *RNASE1* (over expression in MC when compared to PC, see in Fig. 4a) or *TP53INP2* and *RFX4*, down-regulated in the ME group when compared to PC (Fig. 3). A Principal Component Analysis partially supports the previously mentioned results, evidencing also a considerable level of variability between samples (Fig. 5). Nevertheless it also evidences that the majority of the differences observed between both breeds results from the feed restriction treatment applied.

### Gene expression differences between groups

Please refer to Supplementary material 2 , 3, 4, and 5 for a comprehensive description of the expression differences between comparisons. Overall, the *Palmera* breed showed the highest number of DEGs compared to the *Majorera* breed (see also Fig. 3).

A comparison between both control groups (MC vs. PC) showed only twelve differentially expressed (*P* < 0.05) genes (Supplementary material 2). Five up-regulated genes were found in the MC group (*RNASE1*, *POSTN*, *CPXM2*, *LOC102171034* and *CD109*). A total of seven up-regulated genes were found in the PC group (*CIT*, *IL6R*, *GABRB1*, *DPP4*, *RGS4*, *LIPG* and *LOC102184958*).

The *Majorera* mammary gland transcriptome was affected by feed restricition as shown in Supplementary material 3. The genes found to have differential expression were involved in a plethora of biological functions: from RNA processing to energy production, from amino acid transport and nucleotide transport to transcription or signal transduction; and defense mechanisms. Additionally, twenty-four DEGs have been categorized with an unknown function. The DEGs involved in RNA processing (e.g *RNASE1* and *IGFBP6*), signal transduction (e.g. *NGEF*, *PI16* and *SVEP1*) and transcription (e.g. *TP53INP2* and *RFX4*) were mainly down-regulated in the ME group. In this comparison, the majority of the intracellular trafficking, secretion, and vesicular transport pathways were up-regulated in the ME group. The list includes for instance DEGs such as *CXCR4*, *MSR1*, *NKG7* or *CD68*. The extracellular structures had one down-regulated DEG (*OLFML3*) and two up-regulated DEGs (*MT1MMP* and *LGALS3*) in the ME group. Two DEGs associated to cytoskeleton were up-regulated in the MC group compared to the ME group (*ACTG2* and *CNN1*).

Similar to the *Majorera* breed, and as shown in Supplementary material 4, the *Palmera* mammary gland transcriptome was also affected by feed restriction. A total of 99 genes had significantly (*P* < 0.05) differential expression, about 20% more than in the *Majorera* breed. This suggests that the *Palmera* breed is more affected by feed restriction. The list of DEGs include COG terms such as RNA processing, amino acids transport and carbohydrates transport. It also includes translation, transcription, protein turnover, and signal transduction, among others. As in the *Majorera* breed, twenty-four DEGs were detected with unknown roles. The majority of genes involved in lipid, carbohydrate, amino acid and nucleotide transport are up-regulated in the control group.

Finally, the comparison between the two restricted groups (see Supplementary material 5) led to twenty-nine DEGs. These included for instance *ASB11*, *ADAMDEC1* or *S-LZ* (higher expression in the ME group) and *GPD1*, *CTPS1* and *ELOVL6* (higher expression in the PE group).

###  Reverse Transcribed Quantitative PCR validation

**Fig. 6:**
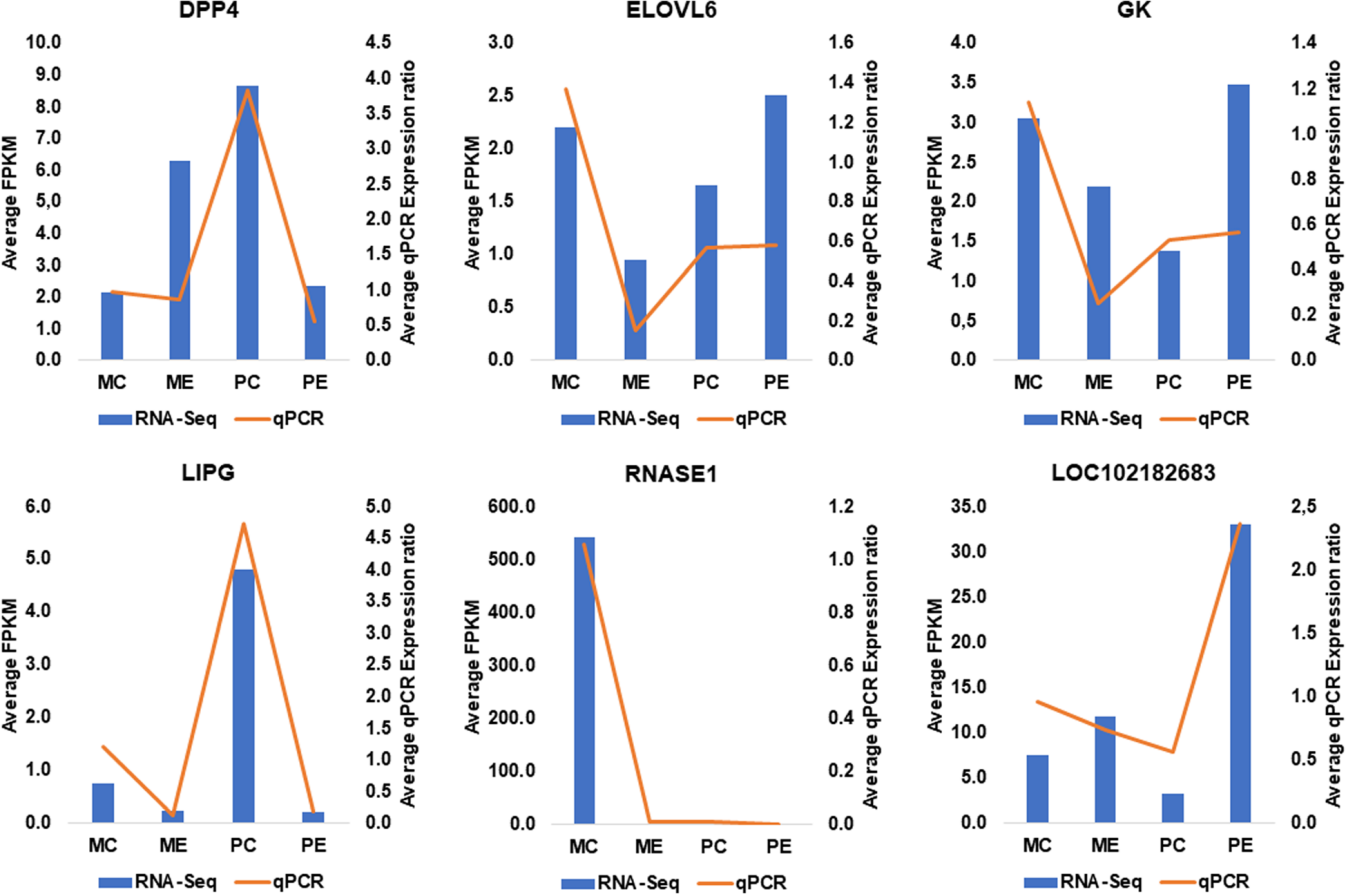
Expression profiles of the six genes (*DPP4*; *ELOVL6*; *GK*; *LIPG*; *RNASE1* and *LOC102182683*) used in the qPCR validation. Bars represent the average of FPKM (Fragments per kilo base per million mapped reads) of biological triplicates for each group obtained using RNA-Seq. Orange lines represent the average of relative gene expression of biological triplicates in each group obtained using RT-qPCR. Y-axis: gene expression values; X-axis: Experimental groups. MC – *Majorera* Control; PC – *Palmera* Control; ME – *Majorera* Restricted and PE – *Palmera* Restricted

The RNA-Seq results were validated using Reverse Transcribed Quantitative PCR. A comparison of the expression levels quantified using RNA-Seq and RT-qPCR is depicted for six randomly chosen genes (*DPP4*; *ELOVL6*; *GK*; *LIPG*; *RNASE1* and *LOC102182683*) (Fig. 6). Results are fully detailed in Supplementary material 8.

**Fig. 7:**
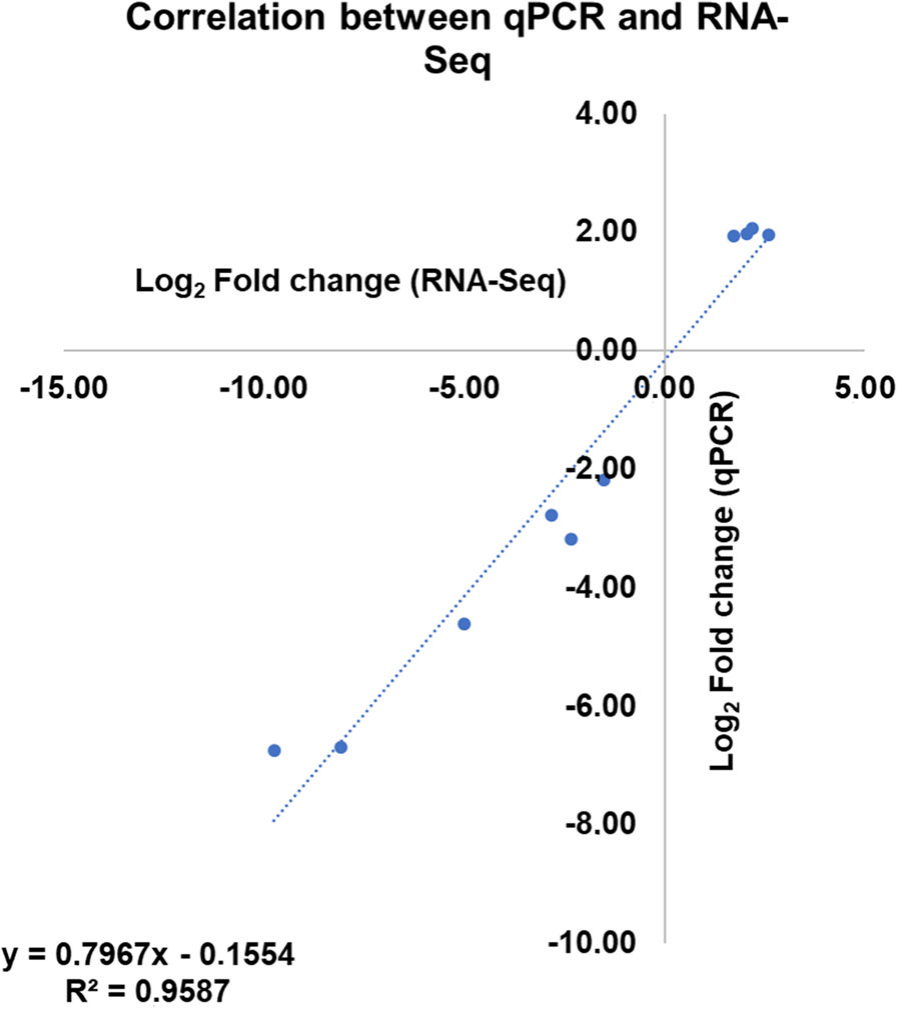
Scatter plot with the Log_2_ fold change observed in RNA-Seq and qPCR for the *RNASE1*, *GK*, *ELOVL6*, *LIPG*, *DPP4* and *LOC102182683* at the comparisons where the genes where considered DE in the RNA-Seq data

A correlation was calculated between the Log_2_ fold change values calculated using the two approaches (qPCR and RNA-Seq) as presented in Fig. 7. A significant positive correlation (R^2^ = 0.9587) was obtained for the mentioned genes. Such correlation supports the accuracy and validation of the RNA-Seq analysis.

## Discussion

In this study, we used a global transcriptomic approach (RNA-Seq) to investigate the molecular mechanisms triggered in the mammary gland as a consequence of weight loss caused by feed restriction in two dairy goat breeds differently adapted to SWL: the tolerant *Majorera* and the susceptible *Palmera*. These breeds have natural differences in size and furthermore have evolved in very different ecosystems which led to differences in their adaptation to nutritional stress [8]. Differences between breeds are mainly based on how specific metabolic pathways were affected by feed restriction, although there are important differences when both control groups are compared. In order to make the results easier to discuss and understand, we have divided this section into different subsections according to the different possible comparisons performed in this study. In these subsections, major results are highlighted, however, in general feed restriction in the two breeds led to a shutdown of cellular mechanisms associated to milk production in agreement with studies by other authors [21].

### Breed effects under control situations

This comparison suggests a high level of similarity between the mammary glands from both breeds when subjected to adequate nutritional levels. This is likely a consequence of a similar genetic background for the ancestors of the two breeds that include both Iberian and African goats that were at some point transported to the Canary Islands [22]. Nevertheless, there are interesting differences.

Genes showing differential expression include: *POSTN* and *RNASE1* (over expression in MC) and *RGS4* (over expression in PC). Gene *POSTN* codes for the protein periostin, which is associated with cell growth in mammary tumors [23, 24], whereas *RNASE1* signals for ribonuclease A family member 1, a protein involved in milk production traits in dairy cattle by regulating protein synthesis and acting as a growth factor in epithelial cells in vitro [25]. Gene *RGS4* codes for regulator of G protein signaling 4. In humans, *RGS4* is involved in the suppression of breast tumor initiation and progression [26], overall indicating a role in the suppression of cell differentiation in sheep. Based on the evidence and considering that the animals were in a similar lactation stage, we can speculate that the MC group had higher mammary gland epithelial growth and differentiation than the PC group. This is likely a consequence of higher milk productions achieved by the *Majorera* breed [8].

### Major pathways affected by weight loss in the *Majorera* mammary gland transcriptome

The *Majorera* mammary gland transcriptome was affected by feed restriction. The genes found to have differential expression are involved in a plethora of biological functions: from RNA processing to energy production, from amino acid transport and nucleotide transport to transcription or signal transduction; and from defense mechanisms to those with an unknown function.

Among the different DEGs involved in RNA processing, *IGFBP6* is an interesting example, and this gene was down-regulated in the ME group. This protein inhibits Insulin Growth Factor which is a protein involved in reducing cell growth and apoptosis protection [27]. In general, these results were expected as a consequence of a slowdown in mammary transcriptional machinery, lowering cell differentiation and proliferation which in turn decreased milk yield. Similar results were observed by Ollier an co-workers [21] in feed deprived dairy goats. On the contrary, Bionaz and co-workers [28] and Palombo and co-workers [29] observed an increased expression of several genes involved in such pathways in the mammary glands of dairy cows and sows transitioning from late gestation to peak lactation, respectively.

We observed a downregulation of DEGs involved in the transport and synthesis of biological compounds likecarbohydrate (*LALBA* or *GK* for instance), lipid (*TM7SF2* or *ELOVL6*) and amino acid (e.g. *TONSL*). These results are a consequence of the reduced metabolic activity of the mammary gland in the ME group when compared to the MC group in accordance with our previous results [8]. Therefore, feed restriction leads to decreases in the synthesis of carbohydrates or lipids in the mammary gland with severe consequences on milk production. Genes involved in these pathways are highly expressed in the mammary gland of dairy goats [30] as well as sheep and cattle [31] and are closely related to milk synthesis and secretion. Again, the pattern of results observed is consistent with studies on mammary gland transcriptomics that contrast for instance animals with different nutritional levels that lead to high or low milk yield [32–35].

The secondary metabolites biosynthesis, transport and catabolism pathway was up- regulated in the ME group as a consequence of weight loss. Two DEGs were described within this pathway: *LOC102175938* and *HSD17B11*. The first, also known as cytochrome b-245 heavy chain [36] is involved in the AGE-RAGE signaling pathway in diabetic complications, being specifically involved in the non-enzymatic oxidation of proteins, lipids and nucleic acids. This gene is also involved in the NOD-like receptor signaling pathway, specifically through the generation of the innate immune response. Gene *HSD17B11* codes for protein hydroxysteroid 17-beta dehydrogenase, and this enzyme is crucial in diverse reproductive pathways in both males and females, regulating intracellular concentrations of inactive and active steroids [37]. It is interesting to point out also one gene in this category, *LOC102191547* also refered to as cytochrome *P450 2F3-like*. This is a gene of the cytochrome P450 (*CYP*) enzyme family involved in steroid metabolism [38]. The over expression of both *HSD17B11* and *LOC102191547* is likely related to cell catabolism and a slowdown of steroid production resulting in the inhibition of mammary gland development [39] This is a common mechanism related to drying off. In this experiment, feed restriction could promote the activation of this mechanism and the decreased milk yield. Nevertheless, results obtained for *LOC102191547* require further investigation.

Regarding the intracellular trafficking and vesicular transport pathways, *CXCR4* or *C-X-C motif chemokine receptor 4* is a gene that is associated specifically to the cytokine-cytokine receptor interaction, leukocyte transendothelial migration and chemokine signaling pathways [40] and is up-regulated in ME. Similarly, *MSR1* or macrophage scavenger receptor 1 is related to phagocytosis [41], *NKG7* codes for natural killer cell granule protein 7 and is involved in the immune response [42] and *CD68* that codes for CD68 molecule protein is associated to the lysosome metabolism. All the genes are therefore involved in the modulation of the inflammatory response. This is likely a consequence of the shutdown and involution of the goat mammary gland, as demonstrated in dairy cows [43]. It seems therefore, that different inflammatory responses associated genes and proteins are up-regulated in the *Majorera* breed because of feed restriction. Considering the defense mechanisms pathway, gene *SVEP1* is one of the various components of the immune response [44], whereas *LOC102187241* codes for protein BPI fold containing family B member 1, a protein of inflammatory response in the upper airway and the progression of non-small cell lung cancer in humans [45]. Such results are difficult to interprete and further studies are needed to better understand these results.

Regarding extracellular structures, gene *OLFML3* codes for olfactomedin like 3, a protein associated with growth, proliferation and neovascularization [46]. Gene *LGALS3* is a gene that codes for galectin 3, a protein over accumulated in mammary epithelial cells during mammary gland involution [47]. Given the involution of the mammary gland in the ME group, the latter results were expected, whereas the first gene being associated to growth is likely to have up-regulation in the MC group. Gene *MT1MMP* codes for matrix metallopeptidase 14, which is associated to mammary gland adipocyte differentiation [48]. It is therefore very surprising that it is up-regulated in the ME group, and it is speculated that this gene may participate in other unknown pathways, as such, further research is still needed.

Regarding genes associated to cytoskeleton, *ACTG2* and *CNN1* code for actin gamma 2 and calponin 1, respectively. Both proteins are involved in smooth muscle contraction [49]. The up-regulation of such genes in the MC group may be a consequence of the higher metabolic activity and gland secretory function maintenance in this group.

Several genes with unknown function were detected. This fact reflects the lack of information available in domestic animal databases, particularly for *Capra hircus*, which limits the research output [16]. Some of these genes coding for proteins involved in different diseases and physiological processes, particularly inflammatory reactions to certain types of human cancer. For instance, *ADAMDEC1*, was up-regulated in the ME group. This gene codes for ADAM like decysin 1, a protein that plays an important role in cancer prevention [50] and intestinal inflammation in humans [51]. Gene *HSD17B11* [37, 52] was also up-regulated in the ME group and was previously addressed. As many of these genes were up-regulated in the ME group, it could be suggested that this increased the expression of genes regulating the immune response during SWL. Nevertheless, such interpretation, albeit interesting, is speculative and needs further investigation.

### Major pathways affected by weight loss in the *Palmera* mammary gland transcriptome

Genes involved in lipid transport, carbohydrate transport, amino acid transport and nucleotide transport are in their majority up-regulated in the control group. Similalry to what was previously discussed for the *Majorera* groups, the *Palmera* mammary gland transcriptome was also affected by feed restriction. The several DEGs include COG terms such as RNA processing, amino acids transport and carbohydrates transport. It includes also translation, transcription, protein turnover, and signal transduction and finally genes with unknown. Function. Some of the results obtained are related to the mammary transcriptional machinery, particularly with the decrease in cell differentiation and proliferation, with a consequent and expectable lower expression in the restricted group.

Contrary to the results obtained in the *Majorera* groups (MC vs. ME), RNA processing and modification (e.g. *F3*), transcription (e.g. *RGS1)* and signal transduction (e.g. *RARRES1*) were up-regulated in the PE group. Overall, these results can be considered as un-expected. Indeed, the PE group showed a decreased activity in mammary transcriptional machinery, with lower levels of cell differentiation and proliferation as described for the whole mammary gland [21] and supported by our previous proteomics analysis [17] and similarly to analogous descriptions by other researchers [53]. Such response seems however to be dependent on the different tissues within the mammary gland, with clear differences between the fat pad and parenchymal tissue, with the latter showing a stabilization of similar COG terms and post-translational modification as a function of lower nutritional levels in Holstein heifers [43]. Our study focuses essentially on the secretory parenchymal tissue and not on the whole mammary gland. As such, further research is needed to enlighten the affected pathways, particularly considering the different subsections of the mammary gland and the variety of different tissues that are part of the organ.

Contrary to the what was observed for the *Majorera* breed, the expression pattern of genes involved in intracellular trafficking, secretion, and vesicular transport pathways are not evident in the *Palmera* groups comparison. Indeed, several DEGs are up-regulated (e.g. *TRPM2* and *CXCR2*) and others down-regulated (*GABRB1* and *RELT*) in the PE group compared to the PC group. Gene *TRPM2* is involved in the NOD-like receptor signaling pathway of the immune response and is involved in cell proliferation [54], whereas *CXCR2* is involved in the modulation of the chemokine signaling pathway and endocytosis. On the contrary, *GABRB1* is involved in neurotransmission [55] and *RELT* in the immune response [56]. The two opposite tendencies in the gene expression could be a consequence of a major disruption of the two pathways. Such divergent tendencies have been recorded in mammary gland involution in dairy cows and water buffalo [57, 58].

The PE group showed an up-regulation of DEGs involved in the defense mechanisms (e.g. *DUSP1*, *DUSP6* and *LOC102190323*). Gene *DUSP* is involved in the suppression of tumorigenic cell proliferation [59], a consequence of decreased cellular proliferation caused by the mammary gland involution. Gene *LOC102190323* is involved in DNA damage response [60]. These results are in agreement with mammary gland transcriptome studies related to involution in goats [21] dairy cows [57] and water buffalo [58].

The extracellular structures pathways showed one DEG down-regulated (*LOC100860938*) and two DGEs up-regulated (*MMP7* and *MMP2*) in the PE group. *LOC100860938* or *COL3A1* codes for collagen type III alpha 1 chain, a protein that is involved in the AGE-RAGE signaling pathway and in turn in cell proliferation [61], rendering these results expectable. Conversely, *MMP2* and *MMP7* code for matrix metallopeptidases. These proteins, particularly *MMP7*, are involved in cellular senescence [62], which agrees with the involution and lowering of different metabolic function in the mammary gland of *Palmera* breed.

The cytoskeleton term in the *Palmera* breed comparison returned *MTAP2* and *TMOD4*, coding respectively for microtubule associated protein 2 (DEG up-regulated in the PE group) and tropomodulin 4 (DEG up-regulated in the PC group). Gene *MTAP2* codes for proteins associated to autophagy in different tissues in the dromedary [63] and particularly during the involution of the bovine mammary gland [64]. Gene *TMOD4* is poorly described and seems to be involved in adipogenesis, myogenesis and the regulation and myofibril assembly in the muscle [39]. Such roles may be extrapolated for the mammary gland, justifying our results.

### Major pathways affected in both feed restricted groups

A comparison between the two reticted groups indicated several pathways with DEGs. These included for instance lipid metabolism, amino acid transport, nucleotide transport, among others. Similarly to the two previous comparisons, many are related to intracellular trafficking and energy metabolism. Gene *GPD1* codes for glycerol-3-phosphate dehydrogenase 1, an enzyme involved in energy production, specifically lipid metabolism [65]. Similar lack of differences in the expression of this gene in the mammary gland of Alpine goats with different levels of lipid supplementation [66] or compared to hay-based diets [67] has also been described. Interestingly, Waldemarson and Karlsson [68] studied the activity of this enzyme in the mammary gland of the Mongolian gerbil (*Meriones unguiculatus*) and found it to be specifically associated to involution of the tissue. The fact that this gene is overexpressed in the PE group likely indicates a lower adaptation to SWL and an earlier onset of the mammary gland involution.

The amino acid transport pathway showed two DEGs overexpressed in the ME group (*CPM* and *ASB11*) compared to the PE group. Gene *CPM* codes for protein carboxypeptidase M, a protein involved in different roles namely hematopoietic cell differentiation, neuronal development with particularly high levels during adipogenesis [69]. Gene *ASB11* codes for protein ankyrin repeat and SOCS box containing 11. This protein has been found to be over-expressed in healthy compared to mastitic and involuting mammary glands in heifers [70]. Carbohydrates transport term only retrieved *S-LZ* gene. This gene codes for protein serum lysozyme, a protein that has long been associated to mammary gland involution [71], specifically through the modulation of antioxidant activity [72]. Therefore, this gene could equally be proposed as a biomarker for SWL tolerance.

Associated to the nucleotide transport pathway, two DEGs were up-regulated (*ENPP1* and *LOC102191092*) and one DEG was down-regulated (*CTPS1*) in the ME group. Gene *ENPP1* codes for protein ectonucleotide pyrophosphatase/phosphodiesterase 1, involved in cell proliferation and differentiation in breast tumors in humans [73]. Gene *LOC102191092* or *CDA* codes for protein cytidine deaminase, a protein related to DNA demethylation and expression of pluripotency genes in bovine cell differentiation [74, 75]. Both findings corroborate our results, highlighting higher cellular activity in the ME group compared to the PE group. On the contrary, *CTPS1* codes for protein CTP synthase 1, a poorly studied protein that has however been associated to lymphocyte proliferation [76]. It could therefore be speculated that its down-regulation in the ME group compared to the PE group that could be associated with increased lymphocyte activity, associated in turn to an advanced involution status of the mammary gland in the latter. Gene *ELOVL6* or ELOVL fatty acid elongase 6 is a gene involved in lipids transport, specifically in fatty acid elongation. Interestingly, this family of genes are down-regulated in the mammary gland of obese rats [77], which supports the results obtained in our study. From such pattern of expression and given the consistency of results, this gene may be suggested as a potential biomarker for SWL tolerance.

The transcription patway retrieved one DEG, while, the translation, ribosomal structure and biogenesis retrieved another DEG and finally the posttranslational modification, protein turnover, chaperones pathways retrieved 5 DEGs. The majority of these genes were up-regulated in the ME group, which was expected, given the fact that the mammary gland of the *Majorera* breed clearly undergoes higher levels of cellular metabolism as previously detailed. On the contrary, *NR4A1* and *LOC102168230*, which code for nuclear receptor subfamily 4 group A member 1 and alpha-2-glycoprotein 1, zinc-binding, respectively had higher expression in the PE group. The first protein is associated to oxidative stress and induced apoptosis in human fibroblasts [78], whereas the latter is related to apoptosis in human breast cancer [79]. The up-regulation of these two genes in the PE group is therefore likely related to a more exacerbated involution-induced apoptosis in these animals.

Associated to intracellular trafficking, secretion, and vesicular transport pathways, two DEGs were down-regulated (*DARC*, *SELP*) and one DEG was up-regulated (*LOC102186944*) in the PE group compared to the ME group. Gene *DARC* codes for atypical chemokine receptor 1, which regulates the inflammatory response [80]. Gene *SELP* codes for selectin P, a protein that mediates and promotes leukocyte adhesion to endothelium, being essential for leukocyte recruitment to an infection site [81]. Gene *LOC102186944* codes for allergen Bos d 2, a poorly studied protein, so far without any specific known described relation to the mammary gland physiology. These results are most likely a consequence of the higher inflammation and leukocyte activity levels in the mammary gland of the PE goats in order to more efficiently manage the mammary gland shutdown. Considering this pattern of expression, higher in the PE group, these genes could be suggested as potential markers for SWL tolerance, although further research is needed.

## Conclusions

As a consequence of weight loss, different genes and pathways show altered expression during the experimental period. The research described in this work, illustrates that different pathways are affected by weight loss in the two dairy goat breeds (*Majorera* and *Palmera*), particularly regarding apoptosis, intracellular trafficking, transcription, as well as lipid and amino acid metabolism. As such, different genes are affected and of possible use in the establishment of markers to SWL, namely those implicated in tolerance (*ENPP1*, *S-LZ*) with higher accumulation in the ME group and susceptibility to SWL (*GPD1*, *CTPS1*, *ELOVL6* and *NR4A1*) with higher accumulation in the PE group. This study unveiled potential candidate genes associated to SWL tolerance, which may impact lactation performance in goats. Based on this, these genes could be used as a reference in future breeding programs for SWL tolerance in dairy goats. Finally, results are likely to be extrapolated to other dairy species such as ewes, water buffaloes and dairy cattle.

## Methods

### Location, animals and nutritional treatments

As described, the animal experiment was performed at the *Universidad de Las Palmas de Gran Canaria* (Arucas, Gran Canaria, Spain) with animals supplied by the *Pico* Research Farm (Valle Guerra, Tenerife, Spain) [8]. Nine *Majorera* and ten *Palmera* goats, 3 lactations with kidding in late February were used [8]. Animals had a body condition score of three (1–5 scale) [8]. Animals were healthy during the whole trial that lasted 22 days [8]. All goats used in this trial were in peak lactation (approximately 50 ± 15 days in milk) [8].

The animals were divided at random into four groups, two per breed: control and feed restricted: MC – *Majorera* control (*n* = 4); ME – *Majorera* restricted (*n* = 5); PC – *Palmera* control (*n* = 6); PE – *Palmera* restricted (*n* = 4) [8]. Animals were milked as described [8, 31, 32]. Milk production per day and BW at the beginning of the trial were 1.60 (± 0.47) l and 45.5 (± 7.74) kg (MC group), 1.68 (± 0.25) l and 50.6 (± 3.64) kg (ME group), 1.03 (± 0.51) l and 32.8 (± 4.91) kg (PC group) and 1.33 (± 0.19) l and 40.6 (± 2.05) kg (PE group) [8]. During the experimental period, animals were housed in two different pens, one for the control groups and one for the underfed groups [8]. A floor space of 1.7 m^2^ per animal was used [8]. Finally, goats had ad libitum access to drinking water [8]. To mimic field conditions of drought-prone regions under SWL, the following nutritional restrictions were used: ME and PE goats were fed on wheat straw and a vitamin-mineral supplement that lead to a BW reduction of 15–20% [8]. On the contrary, MC and PC goats were fed an adequate diet in accordance with lactation requeriments following the INRA’s (*Institut National de la Recherche Agronomique*) procedures sa detailed [8]. The experimental period was 22 days as this was the date when ME and PE goats had a stable 13–15% decrease in relative BW [8]. At day 22, BW and daily milk production were the following: 48.2 kg and 1.99 l (MC group), 44.1 kg and 0.22 l (ME group), 33.9 kg and 1.15 l (PC group) and 35.4 kg and 0.17 l (PE group) as previously described [8].

### Mammary gland sample collection

As described [17, 18], goats were first sedated using an intravenous injection (16 μg/kg of BW) of Xilagesic 20% (Calier, Barcelona, Spain) [17, 18]. The udder was then carefully cleaned and disinfected with a povidone-iodine solution [17, 18]. Prior to biopsy retrieval, local anesthesia was subcutaneously injected (10 mL of 2% lidocaine, *Lidocaína inyectable* 2%, Braun, Barcelona, Spain) to the biopsy collection area [17, 18]. Approximately ten minutes later, a 2 mm incision (left half udder) was made using a scalpel blade (Braun, Tuttingen, Germany) and biopsies collected [17, 18]. The mammary gland was finally sutured and prophylactic treatment administered as described [17, 18].

Biopsy samples (1.5–2 g tissue) were then rinsed in sterile PBS, snap frozen in liquid nitrogen and stored at − 80 °C until further analysis [17, 18]. A total of three samples per group were chosen at random and used in subsequent transcriptomics analysis, similarly to what was previously described [17]. This number of samples is considered as a minimum for a transcriptomics analysis and has been used by other researchers conducting work on the subject [82]. Animal experimental procedures were in accordance with international rules and regulations (European Union procedures on animal experimentation–Directive 2010/63/EU) as described and the protocol was approved by the *Universidad de Las Palmas de Gran Canaria* Ethics committee [17, 18]. Finally, goats used were housed and fed in conditions similar to those used by goat farms in the Canary Islands.

### Tissue mRNA isolation and cDNA synthesis

Total RNA was extracted from the tissue using the E.Z.N.A. Total RNA Kit II (Omega Bio-Tek Inc., Norcross, Georgia, USA). About 30 mg of tissue was finely ground in liquid nitrogen and used with the kit following manufacturer’s instruction to extract total RNA. A DNAse digestion step was carried out after extraction to remove contaminating genomic DNA using an Ambion® TURBO™ DNase (Life Technologies, Carlsbad, CA, U.S.A.) following manufacturer’s instructions. A quantitative and qualitative assessment of the isolated RNA was performed using a NanoDrop® 2000c Spectrophotometer (Thermo Fisher Scientific Inc., Waltham, MA, U.S.A.). Purity was estimated based on the absorbance ratios of the RNA samples at 260/280 nm and 260/230 nm. For both ratios, values were close to 2 (for all samples average 260/280 nm and average 260/230 nm of 1.96 and 1.88 respectively). RNA quality and integrity was also analysed using a RNA Nano Kit Chips from the Agilent 2100 Bioanalyzer System (Agilent Technologies, Santa Clara, CA, U.S.A.). All samples sent to sequencing had a RNA Integrity Number (RIN) above 7. The absence of DNA in RNA treated samples was verified by standard polymerase chain reaction (PCR).

### Next generation sequencing, statistical and bioinformatic analysis

RNA Sequencing was conducted by LC-Sciences (Houston, TX, USA). Twelve mRNA libraries were constructed, 3 biological replicates per condition, following the experimental scheme previously described. Briefly, Poly(A) selection was used and a 100 cycle paired end read was performed using Illumina HiSeq 2500 technology, following the TruSeq® Stranded mRNA Sample Preparation Guide (Illumina). Raw sequencing data can be found in the availability of data and materials section. They were deposited in the NCBI GEO under the accession number GSE151599 and available at: https://www.ncbi.nlm.nih.gov/geo/query/acc.cgi?acc=GSE151599. Data was analyzed using the *Capra hircus* (goat) reference genome, version CHIR_1.0 and available at: https://www.ncbi.nlm.nih.gov/assembly/GCF_000317765.1/. The software default settings were defined using RSEM (v1.2.17, http://deweylab.biostat.wisc.edu/rsem/) to estimate transcript abundance based on fragments per kilobase per transcript per million mapped reads (FPKM), edgeR (v3.2.4) to assess the differentially expressed genes, http://www.bioconductor.org/packages/release/bioc/html/edgeR.html) and Bowtie2 (v2.2.3, http://bowtie-bio.sourceforge.net/bowtie2/index.shtml) used for mapping of the sequences. Categorization of the expressed genes by orthologous groups was performed using Cluster of Orthologous Groups (COG; e-Value 1e-3) within Blast2GO (version 5) with the coding sequence of each transcript. Genes were considered expressed when the raw number counts were at least 100 in at least one sample. Statistical significance was defined as *p*-value < 0.05 (t-test); False Discovery Rate (FDR), calculated using the Benjamini-Hochberg test, below 0.05 and Log2 Fold change (FC) above 1.5 or below − 1.5. The Log_2_ fold change for RNA-Seq was calculated using edgeR. Principal components analysis (PCA) was performed on the expression values obtained for the differentially expressed  genes between at least two  experimental conditions using the software Statistica, version 6 (Statsoft). Before computation, value of fragment per kilobase million (FPKM) were Log_2_ transformed. Heatmaps were obtained via Multiple Experiment Viewer (https://mev.tm4.org; accessed on the 16th of May 2018), Average Linkage Criteria Algorithm and Distance Metric: Pearson, using the Log_2_ FPKM of the DEGs per comparison established.

### Primer design for RT-qPCR experiments

Six candidate genes were selected based on their differential expression results. Primers for candidate genes were constructed: *C. hircus* lipase, endothelial (*LIPG*) (NCBI Gene ID: 102191574), *C. hircus ELOVL* fatty acid elongase 6 (*ELOVL6*) (NCBI Gene ID: 102181762), *C. hircus* glycerol kinase (*GK*) (NCBI Gene ID: 102170215), *C. hircus* dipeptidyl-peptidase 4 (*DPP4*) (NCBI Gene ID: 102184205), *C. hircus* growth-regulated alpha protein-like (*LOC102182683*) (NCBI Gene ID: 102182683) and *C. hircus* ribonuclease, RNase A family, 1 (pancreatic) (*RNASE1*) (NCBI Gene ID: 102174664).

Primer sequences were designed within the coding regions of the target genes from *Capra hircus* genome (CHIR_1.0, annotation release 100) using Primer3 Plus (http://www.bioinformatics.nl/cgi-bin/primer3plus/primer3plus.cgi) and the with selection settings (size 18–22 base pairs (bp); annealing temperature (Tm) 57–63 °C; GC percentage 50–60%) and a PCR product length of 75–150 bp. Primer synthesis was performed by StabVida (ITQB NOVA, Oeiras, Portugal). Primer sequences are detailed in Supplementary material 8.

### Reverse-transcribed quantitative PCR (RT-qPCR)

Messenger RNA was reverse transcribed into cDNA using Oligo(dT) primers included in Promega ImProm-II^tm^ Reverse Transcription System (Promega, Madison, WI, USA) according to the manufacturer’s instructions. One microgram of total RNA per sample was used and three biological replicates per condition (control and restricted groups of both breeds) were reverse transcribed.

The qPCR reactions were performed in a PikoReal Real-Time PCR System (Thermo Fisher Scientific Inc., Waltham, MA, U.S.A.) to measure levels of transcripts of the reference genes. The reaction mixture consisted of 0.2 μl of each 10 mM primer, 5 μL of 2x SYBR® Green Real-time PCR Master Mix (Bio-Rad Laboratories Inc., Hercules, CA, U.S.A.), 1 ng of the each reverse transcribed cDNA and autoclaved Milli-Q water to a final volume of 10 μL. Reactions were performed in duplicate with a non-template control, on 96-well optical reaction plates. The qPCR programme consisted of an initial incubation step at 95 °C for five min, amplifications were performed for 40 cycles with the following cycle profile: a denaturing step at 95 °C for 10 s followed by an annealing step at 60 °C for 30 s. The specificity of the qPCR products was confirmed through a melting temperature analysis from 55 °C to 95 °C in 0.2 °C intervals. The melting curve obtained was analyzed to confirm the presence of a single gene-specific peak and the total absence of primer-dimers. The GenEx Pro software (GenEx Pro 4.3.7, MultiD Analyses AB, Göteborg, Sweden), which includes in its pipeline for data analysis the NormFinder [83] and geNorm [84] algorithm, was used to identify the most stable reference genes.

For each primer pair the efficiency of the reaction and slope values were determined using LinReg Software (v2013.0) based on previously published work [85]. All data, including the cycle threshold (Cq) obtained through LinReg Software were used for the delta Ct method [86]. Relative gene expression of candidate genes was calculated using the delta Ct method with two reference genes beta-actin (*ACTB*) [87, 88] and elongation factor 1 alpha 1 (*EEF1A1*). Please refer to supplementary material 8 for additional details on RT-qPCR results.

## Supplementary information

**Additional file 1.** Raw read counts for all genes and all samples in the study.

**Additional file 2. **List of differentially expressed genes between *Majorera* Control vs. *Palmera* Control. The Log_2_ Fold Change (Log_2_FC), Log_2_ of counts per million (Log_2_Counts), calculated *P*-value and FDR is shown. Raw reads for each gene in each sample are described: *Majorera* Control (MC1, MC2 and MC3) and *Palmera* Control (PC1, PC2 and PC3).

**Additional file 3. **List of differentially expressed genes between *Majorera* Control vs. *Majorera* Restricted. The Log_2_ Fold Change (Log_2_FC), Log_2_ of counts per million (Log_2_Counts), calculated *P*-value and FDR is shown. Raw reads for each gene in each sample are described: *Majorera* Control (MC1, MC2 and MC3) and *Majorera* Restricted (ME1, ME2 and ME3).

**Additional file 4. **List of differentially expressed genes between *Palmera* Control vs. *Palmera* Restricted. The Log_2_ Fold Change (Log_2_FC), Log_2_ of counts per million (Log_2_Counts), calculated *P*-value and FDR is shown. Raw reads for each gene in each sample are described: *Palmera* Control (PC1, PC2 and PC3) and *Palmera* Restricted (PE1, PE2 and PE3).

**Additional file 5. **- List of differentially expressed genes between *Majorera* Restricted vs. *Palmera* Restricted. The Log_2_ Fold Change (Log_2_FC), Log_2_ of counts per million (Log_2_Counts), calculated P-value and FDR is shown. Raw reads for each gene in each sample are described: *Majorera* Restricted (ME1, ME2 and ME3) and *Palmera* Restricted (PE1, PE2 and PE3).

**Additional file 6.** All genes expressed in the study, with respective FPKM (Fragments per kilo base per million mapped reads) value in each sample.

**Additional file 7.** Tables summarizing the orthologous groups for each DE gene found in the main comparisons established (obtained via Blast2GO, Cluster of orthologs e-Value 1e-3).

**Additional file 8.** – Details of the primers built for reference and candidate genes to be used in RT-qPCR, expression values from RNA-Seq and qPCR used for expression profiling and correlation analysis.

**Additional file 9. **– Output of the edgeR analysis for the *Majorera* Control and *Majorera* Restricted comparison. The Log_2_ Fold Change (Log_2_FC), Log_2_ of counts per million (Log_2_Counts), calculated *P*-value and FDR is shown. Raw reads for each gene in each sample are described: *Majorera* Control (MC1, MC2 and MC3) and *Majorera* Restricted (ME1, ME2 and ME3). NCBI gene and transcripts IDs are provided. Only genes with Log_2_FC > 0.5 or Log_2_FC < 0.5 are provided.

**Additional file 10. **– Output of the edgeR analysis for the *Palmera* Control and *Palmera* Restricted comparison. The Log_2_ Fold Change (Log_2_FC), Log_2_ of counts per million (Log_2_Counts), calculated P-value and FDR is shown. Raw reads for each gene in each sample are described: *Palmera* Control (PC1, PC2 and PC3) and *Palmera* Restricted (PE1, PE2 and PE3). NCBI gene and transcripts IDs are provided. Only genes with Log_2_FC > 0.5 or Log_2_FC < 0.5 are provided.

**Additional file 11 **– Output of the edgeR analysis for the *Majorera* Control and *Palmera* Control groups comparison. The Log_2_ Fold Change (Log_2_FC), Log_2_ of counts per million (Log_2_Counts), calculated P-value and FDR is shown. Raw reads for each gene in each sample are described: *Majorera* Control (MC1, MC2 and MC3) and *Palmera* Control (PC1, PC2 and PC3). NCBI gene and transcripts IDs are provided. Only genes with Log_2_FC > 0.5 or Log_2_FC < 0.5 are provided.

**Additional file 12 **– Output of the edgeR analysis for the *Majorera* Restricted and *Palmera* Restricted groups comparison. The Log_2_ Fold Change (Log_2_FC), Log_2_ of counts per million (Log_2_Counts), calculated P-value and FDR is shown. Raw reads for each gene in each sample are described: *Majorera* Restricted (ME1, ME2 and ME3) and *Palmera* Restricted (PE1, PE2 and PE3). NCBI gene and transcript IDs are provided. Only genes with Log_2_FC > 0.5 or Log_2_FC < 0.5 are provided.

## Data Availability

Data was analyzed using the *Capra hircus* (goat) reference genome, by the International Goat Genome Consortium, version CHIR_1.0 and available at: https://www.ncbi.nlm.nih.gov/assembly/GCF_000317765.1/. All data in this study are included in the article and additional files. Raw read values for all samples obtained using RNA-Seq are available in Supplementary Material 1, the FPKM values for all genes are available in Supplementary Material 6, edgeR analysis outputs are available in supplementary material 9, 10, 11 and 12. Raw sequencing data were deposited in the NCBI GEO under the accession number GSE151599 (https://www.ncbi.nlm.nih.gov/geo/query/acc.cgi?acc=GSE151599), including the samples Majorera Control 1 (GSM4586432), Majorera Control 2 (GSM4586433), Majorera Control 3 (GSM4586434), Majorera Restricted 1 (GSM4586437), Majorera Restricted 2 (GSM4586435), Majorera Restricted 3 (GSM4586436), Palmera Control 1 (GSM4586438), Palmera Control 2 (GSM4586439), Palmera Control 3 (GSM4586440), Palmera Restricted 1 (GSM4586441), Palmera Restricted 2 (GSM4586442) and Palmera Restricted 3 (GSM4586443).

## Abbreviations

*ACTB*: Actin beta
*ACTG2*: Actin gamma 2
*ADAMDEC1*: ADAM like decysin 1
*AGE-RAGE*: Advanced glycation end products - receptor for advanced glycation end products
*ASB11*: Ankyrin repeat and SOCS box containing 11
BW: Liveweight
*CD68*: CD68 molecule
*CDA*: Cytidine deaminase
*CNN1*: Calponin 1
COG: Cluster of Gene Ontology
*CPM*: Carboxypeptidase M
*CSN1S2*: Alpha 2-casein
*CSN2*: Casein beta
*CSN3*: Casein kappa
*CTPS1*: CTP synthase 1
*CXCR2*: C-X-C motif chemokine receptor 2
*CXCR4*: C-X-C motif chemokine receptor 4
*CYP*: Cytochrome P450
*DARC*: Atypical chemokine receptor 1
DEG: Differentially Expressed Genes
*DPP4*: Dipeptidyl peptidase 4
*DUSP1*: Dual specificity phosphatase 1
*DUSP6*: Dual specificity phosphatase 6
*EEF1A1*: Eukaryotic translation elongation factor 1 alpha 1
*ELOVL6*: ELOVL fatty acid elongase 6
*ENPP1*: Ectonucleotide pyrophosphatase/phosphodiesterase 1
FC: Fold Change
FDR: False Discovery Rate
FPKM: Fragments per kilobase per million mapped reads
*GABRB1*: Gamma-aminobutyric acid type A receptor subunit beta1
*GK*: Glycerol kinase
*GPD1*: Glycerol-3-phosphate dehydrogenase 1
HCl: Chloride Hydrogen
*HSD17B11*: Hydroxysteroid 17-beta dehydrogenase 11
*IGFBP6*: Insulin like growth factor binding protein 6
*LALBA*: Lactalbumin alpha
*LGALS3*: Galectin 3
*LIPG*: Lipase G, endothelial type
MC: *Majorera* Control
ME: *Majorera* Restricted
*MMP2*: Matrix metallopeptidase 2
*MMP7*: Matrix metallopeptidase 7
*MSR1*: Macrophage scavenger receptor 1
*MT1MMP*: Matrix metallopeptidase 14
*MTAP2*: Microtubule associated protein 2
*NGEF*: Neuronal guanine nucleotide exchange factor
*NKG7*: Natural killer cell granule protein 7
*NR4A1*: Nuclear receptor subfamily 4 group A member 1
*OLFML3*: Olfactomedin like 3
*PAEP*: Progestagen-associated endometrial protein
PC: *Palmera* Control
PCA: Principal Component Analysis
PE: *Palmera* Restricted
*PI16*: Peptidase inhibitor 16
*POSTN*: Periostin
*RARRES1*: Retinoic acid receptor responder 1
*RELT*: RELT TNF receptor
*RFX4*: Regulatory factor X4
*RGS1*: Regulator of G protein signaling 1
*RGS4*: Regulator of G protein signaling 4
*RNASE1*: Ribonuclease pancreatic-like
RNA-Seq: RNA sequencing
*SELP*: Selectin P
S-LZ: Serum lysozyme
*SPTLC3*: Serine palmitoyltransferase long chain base subunit 3
*SVEP1*: Sushi, von Willebrand factor type A, EGF and pentraxin domain containing 1
SWL: Seasonal weight loss
*TM7SF2*: Transmembrane 7 superfamily member 2
*TMOD4*: Tropomodulin 4
*TONSL*: Tonsoku like, DNA repair protein
*TP53INP2*: Tumor protein p53 inducible nuclear protein 2
*TRPM2*: Transient receptor potential cation channel subfamily M member 2
